# Refining the characterization of residual function in hypertrophic cardiomyopathy through remote segment 4D strain analysis

**DOI:** 10.1186/1532-429X-18-S1-P234

**Published:** 2016-01-27

**Authors:** Alessandro Satriano, Nita Guron, Yoko Mikami, Naeem Merchant, Carmen P Lydell, Andrew G Howarth, Nowell M Fine, James A White, Bobak Heydari

**Affiliations:** 1 Stephenson Cardiac Imaging Centre, Calgary, AB Canada; 2 grid.22072.350000000419367697Division of Cardiology, School of Medicine, University of Calgary, Calgary, AB Canada; 3 grid.22072.350000000419367697Department of Diagnostic Imaging, University of Calgary, Calgary, AB Canada

## Background

Quantitative assessment of late gadolinium enhancement (LGE) by cardiovascular magnetic resonance imaging (CMR) has been associated with an increased risk of sudden cardiac death (SCD) in patients with hypertrophic cardiomyopathy (HCM). However, patients with lesser degrees of LGE may still remain at high risk of adverse cardiac events due to the diffuse pathophysiology of HCM. Non-invasive characterization of the degree of biomechanical strain within non-enhanced myocardium may be a novel marker of disease in patients with HCM.

## Methods

Forty-one consecutive patients with HCM and 40 healthy controls underwent CMR at 3T (Skyra, Siemens, Germany). 4D strain analysis was performed using GIUSEPPE, an in-house software that allows tracking of a 3D patient-specific ventricular mesh across the cardiac cycle relying on a 4D velocity field reconstructed from feature tracking of routinely acquired, long and short axis cine SSFP views. Segments with LGE (defined as >6SD beyond remote myocardium) were excluded to define mean global principal, radial, circumferential, and longitudinal strain values. HCM patients were categorized into those with total volume of LGE ≥15% or <15%.

## Results

**Figure 1:**
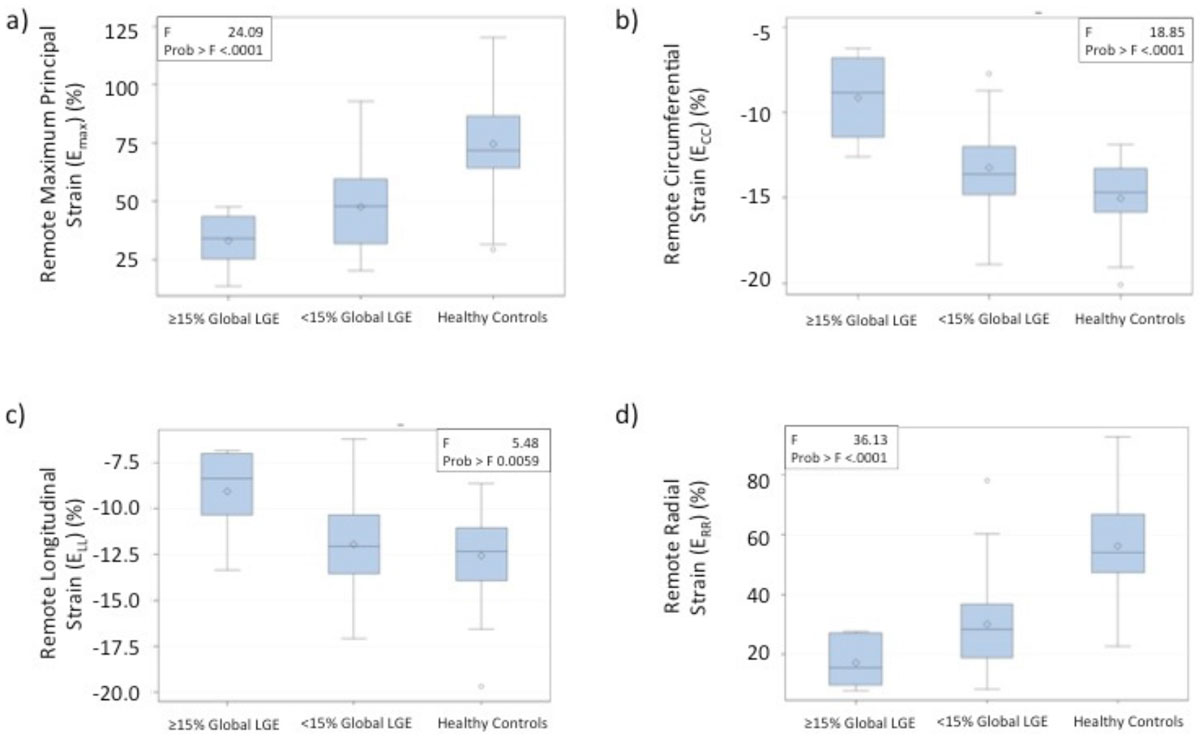
**Boxplots representing the distributions of mean Maximum Principal (a), Circumferential (b), Longitudinal (c) and Radial (d) strain in remote tissue for HCM patients characterized by global LGE >=15%, <15% and for healthy controls**.

**Figure 2:**
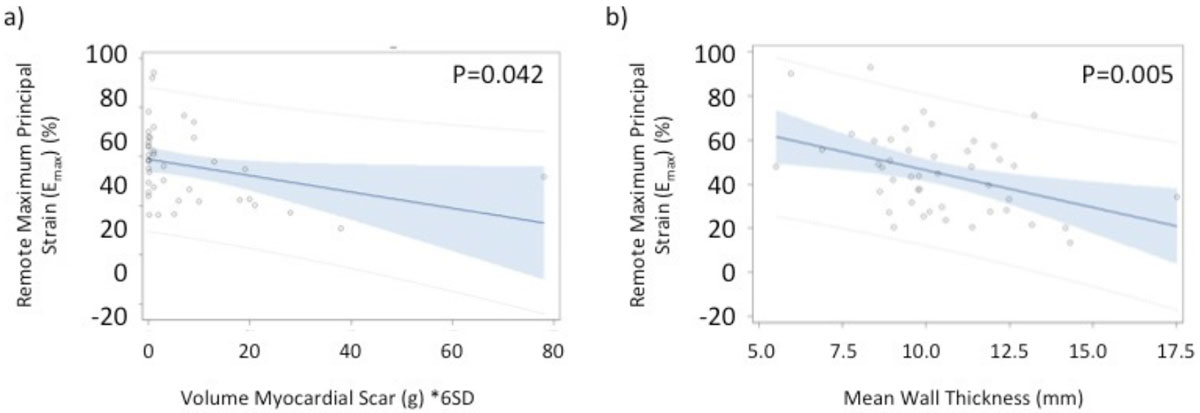
**Linear regression of remote maximum principal strain vs. Volume of myocardial scar as estimated with a 6SD threshold (a) and vs. mean wall thickness (b)**.

Mean age of the HCM patients was 54.6 ± 12.5 years (36% female) with mean LVEF of 76.4 ± 9.0%. Mean percentage of global LGE was 5.5 ± 9.7% for the total cohort. All measures of 4D strain were significantly lower within the non-enhanced myocardium of HCM patients as compared with healthy controls (Figure 1). Furthermore, 4D principal, circumferential, and radial strains within non-enhanced myocardium were significantly reduced in those patients with ≥15% LGE, as compared to those with <15% (Figure 1). Linear regression analysis revealed a significant association between the degree of 4D principal strain within the non-enhanced myocardium of HCM patients and both volume of myocardial scar and mean myocardial wall thickness (Figure 2).

## Conclusions

This study demonstrated significantly reduced 4D strain amplitude within non-enhanced segments of HCM patients as compared to normal controls. Furthermore, those patients with greater degrees of global LGE (≥15%) had significantly reduced parameters of 4D strain. Further studies using this technique for the assessment of regional 4D biomechanical strain to prognosticate sudden cardiac death and congestive heart failure within HCM patients are warranted.

